# Chimeric-antigen receptor T (CAR-T) cell therapy for solid tumors: challenges and opportunities

**DOI:** 10.18632/oncotarget.19361

**Published:** 2017-07-18

**Authors:** An-Liang Xia, Xiao-Chen Wang, Yi-Jun Lu, Xiao-Jie Lu, Beicheng Sun

**Affiliations:** ^1^ Liver Transplantation Center of the First Affiliated Hospital and Collaborative Innovation Center For Cancer Personalized Medicine, Nanjing Medical University, Nanjing, Jiangsu Province 210029, P.R. China

**Keywords:** chimeric antigen receptor, T cells, cancer, adoptive cell transfer, tumor microenvironment

## Abstract

Chimeric antigen receptor (CAR)-engineered T cells (CAR-T cells) have been shown to have unprecedented efficacy in B cell malignancies, most notably in B cell acute lymphoblastic leukemia (B-ALL) with up to a 90% complete remission rate using anti-CD19 CAR-T cells. However, CAR T-cell therapy for solid tumors currently is faced with numerous challenges such as physical barriers, the immunosuppressive tumor microenvironment and the specificity and safety. The clinical results in solid tumors have been much less encouraging, with multiple cases of toxicity and a lack of therapeutic response. In this review, we will discuss the current stats and challenges of CAR-T cell therapy for solid tumors, and propose possibl e solutions and future perspectives.

## INTRODUCTION

**Figure 1:**
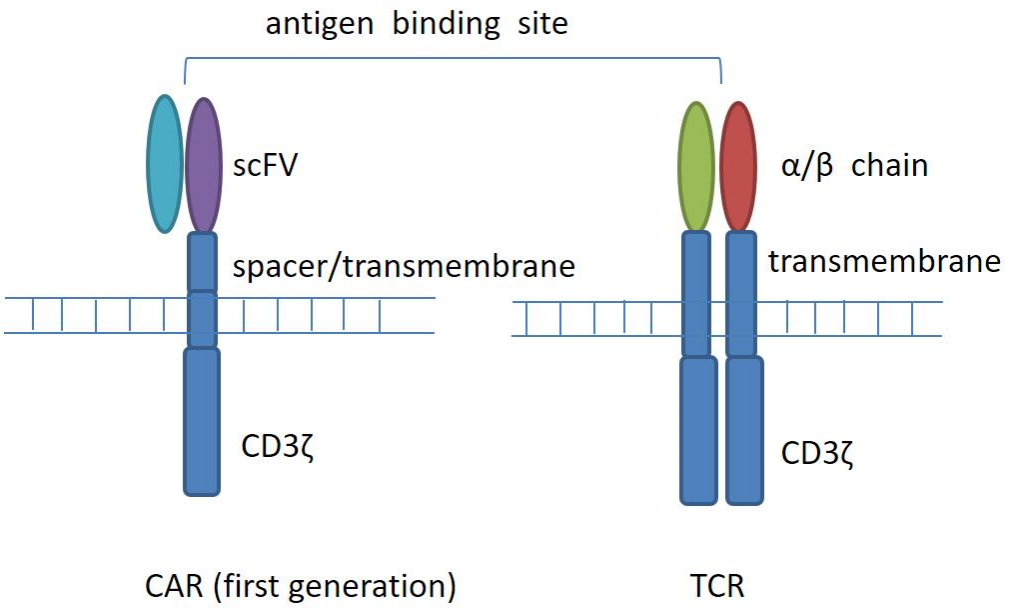
T-cell receptor (TCR) and chimeric antigen receptor (CAR) structure CARs are composed of a membrane-distal single-chain variable fragment (scFv), a spacer domain, a transmembrane domain, and intracellular signaling/activation domain. CARs recognize surface antigens in a non-MHC manner. T-cell receptors are composed of two separate proteins, the alpha (α) and beta (β) chains. TCR identifies intracellular or extracellular proteins that are presented as peptides by MHC molecules.

Chimeric antigen receptor (CAR) T cells refer to T cells that are genetically modified to express chimeric antigen receptors. Four components comprise CAR, an extracellular target binding domain named single-chain variable fragment (scFv), a spacer domain, a transmembrane domain, and intracellular signaling/activation domain (Figure 1). Compared with T cell receptor (TCR) modified cells, CAR T cells have the capacity to recognize cell surface tumor antigens in an HLA-independent fashion, leading to antigen-specific T cell activation, proliferation, and cytokine production, and combating tumor. CARs recognize a range of antigens in a non-MHC manner, thus expanding the range of clinical application.

According to the gene transfer method, T cells can be permanently or transiently modified to express CARs. Vectors derived from retroviruses or lentiviruses are integrated into the host genome by DNA to produce stable transduction. In contrast, RNA insertion allows transient expression without permanently modifying the host cell genome. Most of the clinical trials performed today use unselected *in vitro* amplification of T cells obtained from peripheral blood mononuclear cells (PBMC). In order to produce enough engineered T cells, amplification is achieved by *in vitro* cell culture systems. Different cell culture systems produce a wide variety of T cell subsets consisting of different proportions of naive, effector and memory T cells. Since this composition may be important for replication and persistence, some teams have introduced a selection step to enrich for the central memory T cells [1, 2]. Recently, various methods have been developed for the isolation of defined T cell subsets under good manufacturing (GMP) conditions in order to better control the phenotype of the transferred T cells [3].

Researchers have studied the engineering of T cells to express chimeric antigen receptors that target tumor antigens for more than 20 years [4, 5]. The first clinical research at the University of Pennsylvania achieved two complete responses in three patients with refractory advanced CLL using anti-CD19 CAR T cells [6, 7]. And four years later, an overall response rate of 57 % was demonstrated in a study by the same group [8]. Recent studies have shown that the success of CAR T cells in treating hematological malignancies is remarkable, particularly in acute lymphoblastic leukemia (ALL) with the complete remission rate of 90% and sustained remissions of up to 2 years [9]. This impressive result leads to a large number of clinical trials of CAR T cells aiming at multiple hematological antigens, such as CD19 [10–12], CD20 [13, 14] CD22 [15] and CD30 [16]. In addition, compared with unselected T cells and CD8 or CD4 T cells alone, CAR T cells consisting of CD4 T cells derived from the naive CD4 T cell pool and CD8 T cells derived from central memory CD8 T cells at a 1:1 ratio, showed superior efficiency in mouse lymphoma model [1]. However, in all trials, the anti-tumor effect correlated with the persistence and proliferation of CAR T cells in the peripheral blood of the patients. Poor *in vivo* expansion and persistence limited clinical progress after engineered T cells infusion [17–22].

CD19 is recognized as a target for immunotherapy in B cell malignancies because of its limited expression on mature B cells rather than other hematopoietic cells or non-hematopoietic tissues. Objective regression was achieved in patients with acute lymphoblastic leukemia (ALL), chronic lymphocytic leukemia (CLL) and other types of B cell lymphoma *via* application of CAR T cells which are redirected against CD19 [8, 11, 12, 23]. Compared with conventional therapies such as radiotherapy or chemotherapy, CAR T cell trials targeting CD19 exhibited a favorable and lasting clinical outcome. To date, a majority of early-phase trials have been and are currently being performed to treat B cell malignancies, with only a minority of trials targeting solid cancer, and the most successful CARs have been those specific for CD19 on B cell malignancies. Unfortunately, the clinical results in solid tumors have been much less encouraging, with multiple cases of toxicity and/or a lack of therapeutic response [18, 19, 24, 25]. In this review, we will mainly discuss the challenges and possible solutions of CAR-T cell therapy for solid tumors.

## CAR-T CELL THERAPY FOR SOLID TUMORS

**Figure 2:**
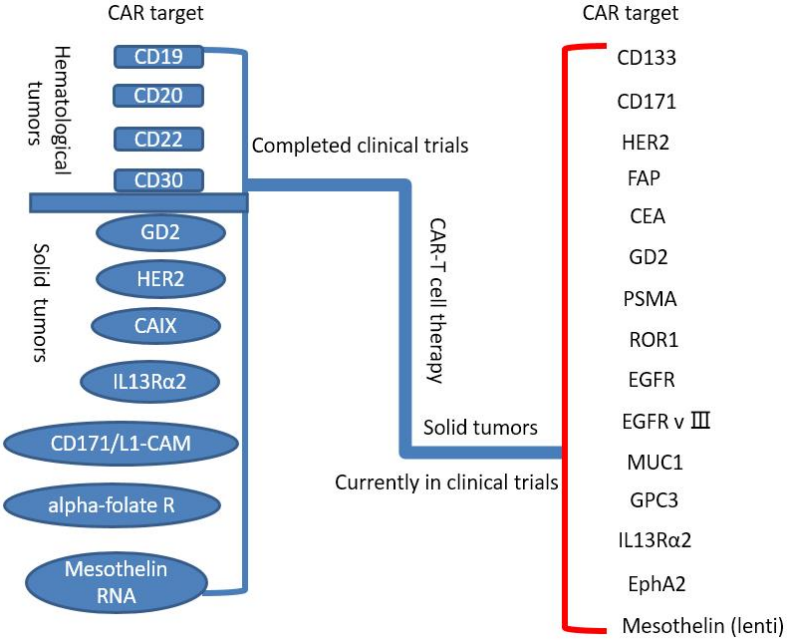
Completed and ongoing CAR-T cell therapy clinical trials (per ClinicalTrials.gov)

To date, CAR T cells have made great success in treatment of hematologic malignancies, such as allogeneic CD19-CAR-T cell in B cell malignancies [26]. On this basis, a rising number of trials have been done to investigate the value of CAR T cell therapy for solid tumors (Table 1, Figure 2), for instance, the breast carcinoma, the sarcoma, the neuroblastoma, etc. Some quantity of trials fix their sight on surface proteins and integrin, involving carcinoembryonic antigen (CEA) for colorectal adenocarcinoma [27], fibroblast activation protein (FAP) for malignant pleural mesothelioma [28], the diganglioside GD2 for neuroblastoma and osteosarcoma [29], human epidermal growth factor receptor 2 (HER2) for HER2-positive sarcoma [30], mesothelin for pancreatic cancer [31], interleukin 13 receptor α (IL-13Rα) for glioma [32], aberrant αvβ6 integrin for pancreatic tumor [33] and so on. Nevertheless, the results of trials are barely satisfactory. Some reported trials applied GD2-specific CAR T cells for neuroblastoma (insufficient working time of CAR T cells with some evidence of antineoplastic effects) [34], HER2 CAR T cells for HER2-positive sarcoma (3 of 17 patients with tumor removed) [30], epidermal growth factor receptor (EGFR) CAR T cells for non-small cell lung cancer (2 of 11 patients with partial responses and 5 of 11 with stable disease) [35], and anti-CEA CAR T cells for CEA+ Liver Metastases(One patient alive with stable disease and 5 patients dead of progressive disease) [36].

**Table 1:** Examples of CAR-T cell clinical trials

Target	Type of cancer	ID
CD133	Hepatocellular carcinoma, breast cancer	NCT02541370
CD171	Neuroblastoma, ganglioneuroblastoma	NCT02311621
HER2	Gastric cancer, Advanced sarcoma	NCT01935843 and NCT00902044
CEA	gastric cancer, pancreatic cancer Colorectal adenocarcinoma	NCT02349724 and NCT00004178
FAP	Malignant pleural mesothelioma	NCT01722149
GD2	Neuroblastoma, Ewing's sarcoma, osteosarcoma and melanoma	NCT01822652 and NCT02107963
MUC1	Hepatocellular carcinoma, NSCLC, pancreatic cancer and triple-negative invasive breast cancer	NCT02617134 and NCT02587689
IL13Rα2	Glioma	NCT02208362
EGFR	Advanced glioma, EGFR positive solid tumors	NCT02331693 and NCT01869166
EGFR vIII	Glioblastoma	NCT02209376 and NCT01454596
mesothelin	Pancreatic cancer	NCT02706782

Unfortunately, a large amount of the clinical results have not met our expectation. CAR T cell therapy for solid tumors is inferior to that for hematologic malignancies. What leads to the less rewarding outcomes is the unavoidable factors when we use CAR T cells to treat solid tumors. Large quantities of differences between hematological malignancies and solid tumors are supposed to be taken into consideration. Firstly, hematological malignancies are usually disseminated, but one solid tumor is often a concrete mass located at one organ at early stage. Secondly, the target antigens of hematological malignancies tend to be homogeneous, with most malignant tumors carrying target antigens. On the contrary, target antigens expressed on solid tumors are heterogenous mostly, differing not only between different solid tumors but also between the primary and metastatic stages of the same tumor. Moreover, solid tumors are surrounded by physical immunosuppressive factors preventing adoptively transferred cells from migrating to solid tumors, whereas hematological malignancies lack these factors.

Besides the differences referred above, many additional factors leading to the less encouraging results are necessary to be taken into account. Reaching the solid tumors under the guidance of the correct chemotactic signals from the blood is the first intractable hurdle. Some unavoidable aspects make this step difficult, including the interference of chemokines secreting by solid tumors, physical barriers constructed by surrounding stroma and immune cells, and even the deviant vasculature approach to tumors. Unlike the homogenous antigens secreted by hematological malignancies, solid tumors express heterogenous antigens targeted by CAR T cells, which make them more difficult to meet. As is well known, solid tumors secrete a variety of chemokines like CXCL5 and CXCR2, which compose the signal path preventing T cells from migrating to the advanced prostate cancer [37]. The immunosuppressive tumor microenvironment (TME) recruits immunosuppressive cells such as myeloid cells and fibroblasts, which constitute the fibrotic extracellular matrix surrounding the tumor and inhibit the infiltration of T cells into the tumor [38].

Moreover, the adverse TME is composed of diverse molecular and cellular elements, making CAR T cells dysfunctional before long. For example, oxidative stress, nutritional short, acidic pH, and oxygen absence threaten the efficiency of CAR T cells. The secretion of immunosuppressive cytokines, namely TGF-βand IL-10; suppressive immune cells, such as myeloid-derived suppressor cells (MDSCs), regulatory T cells (Tregs) and tumor-associated macrophages (TAMs) or neutrophils (TANs); and checkpoint inhibitory proteins including PD-L1 damage efficient anti-tumor function of the CAR T cells. Also, due to its potential immunogenicity and toxicity, CART cells themselves may influence the surrounding immune environment.

Finally, the nonnegligible adverse effect of the CAR T cells therapy for solid tumors are supposed to be envisaged. Besides the tumor, the CAR T cells attack normal tissues of the body involving heart, lung, brain and liver owing to target antigens also expressed in these important organs.

As mentioned above, the therapy of CAR T cells is promising, but there are still a lot of unsolved tissues.

## CHALLENGES AND POSSIBLE SOLUTIONS FOR CAR-T CELL THERAPY FOR SOLID TUMORS

**Figure 3:**
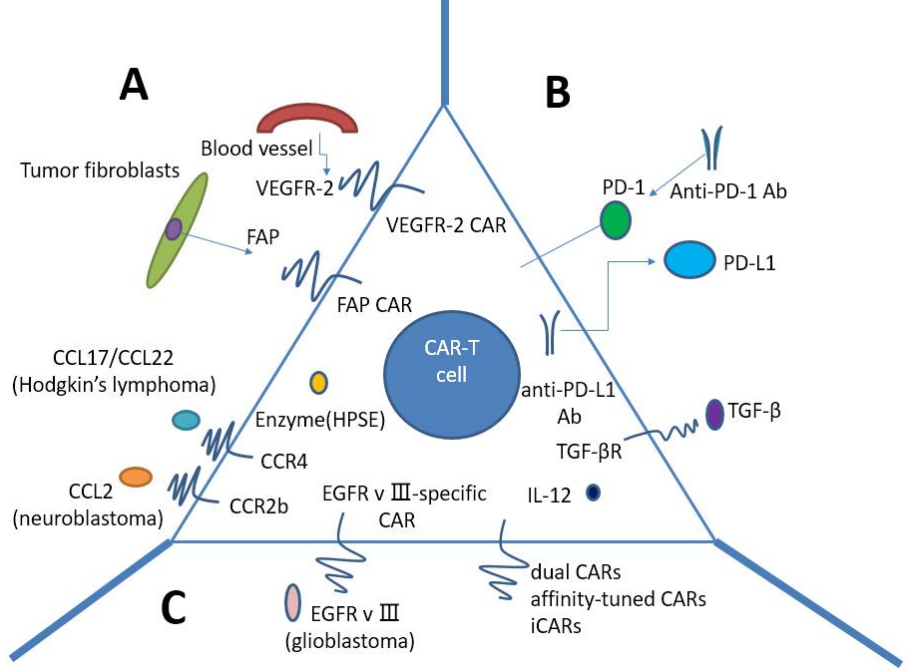
Strategies to improve CAR T-cells for treatment of solid tumors **A.** Penetrating physical barriers and homing to the tumors. a. Targeting tumor stroma or vasculature: FAP-CAR T cells can reduce tumor fibroblast numbers and thus inhibit tumor growth. b. Secreting enzyme: HPSE can disintegrate heparan sulphate proteoglycans, which mainly constitute ECM. c. Expressing chemokine receptor (CCR4, CCR2b): we may genetically modulate CAR T cells to express the chemokine receptor(s) matching the tumor chemokine, allowing a lot of T cells to home to the tumors. **B** Immunosuppressive tumor microenvironment. a. CAR T cells capable of secreting anti-PD-L1 antibodies: secretion of anti-PD-L1 antibodies from CAR T cells can also enable human NK cells to migrate into the tumor site *in vivo*. b. PD-1 blockade using antihuman antibodies can potently enhance CAR T-cell therapy. c. IL-10/TGF-β receptor blockade. d. armored-CARs and TRUCKS capable of secreting pro-inflammatory cytokines (IL-12 exhibits enhanced antitumor efficacy) **C.** Specificity and safety of CAR-T cells. a. EGFRvIII-specific CAR: the only truly tumor-specific antigen for CAR is EGFRvIII that is completely restricted to human cancer (most frequently observed in glioblastoma). b. Dual CARs (two different CARs): CAR number one contains the CD3ζ signaling domain, activating T cell function, whereas CAR number two provides the costimulation signaling function by CD28 and/or CD137. c. Affinity-tuned CARs. d. iCARs: inhibitory CARs, specific for the antigen expressed on normal cells but not on tumor cells, will keep the normal cells from a CAR-T cell-mediated attack due to negative signaling endowed by iCARs. CAR: chimeric antigen receptor; FAP: fibroblast activation protein; ECM: extracellular matrix; HPSE: heparanase; iCARs: inhibitory CARs; TRUCKs: T cells redirected for universal cytokine killing.

CAR T-cell therapy for solid tumors currently is faced with numerous challenges (Figure 3). Firstly, physical barriers hinder CAR T cells sufficiently infiltrating the tumors, such as the surrounding stroma. Secondly, CAR T cells must be confronted with immunosuppressive tumor microenvironment (TME) after reaching the tumor. Last but not least, what we should consider is the specificity and safety of CAR T cells.

### Penetrating physical barriers and homing to the tumors

Physical barrier is the first of many hurdles that CAR T cells encounter before entering the immunosuppressive TME, preventing efficient infiltration into the tumors (Figure 3A). Tumor fibroblasts and myeloid cells are in favor of the development of fibrotic extracellular matrix (ECM), which may impede T cell penetration. Wang LC et al. have demonstrated the enhanced CAR T cell function by using FAP-CAR T cells in animal models, reducing tumor fibroblast numbers and thus inhibiting tumor growth [39]. One enzyme named heparanase (HPSE) can disintegrate heparan sulphate proteoglycans, which mainly constitute ECM. However, we found that *in vitro*-cultured T cells do not express HPSE. We therefore engineered CAR T cells to express HPSE in order to degrade ECM and overcome the physical barriers, thus improving T-cell infiltration and antitumor activity. Caruana I et al. have showed this success [40].

In addition, compared to hematologic malignancies, solid tumors can secrete some chemokines preventing T cells from migrating to the tumors, such as CXCL12 and CXCL5 [37, 41]. Furthermore, owing to the mismatch between chemokine receptors expression on T cells and tumor chemokine signature, T lymphocytes can hardly migrate into the tumor, and thus lose the immune function against cancer [42]. Given this, we may genetically modulate CAR T cells to express the chemokine receptor(s) matching the tumor chemokine, allowing a lot of T cells to home to the tumors. Also, using vasculature-targeted CAR T cells maybe improve the delivery rate. Actually, this method has been demonstrated in Hodgkin's lymphoma using CCR4-bearing CAR T cells [43], and in mesothelioma and neuroblastoma xenografts using CCR2b-bearing CAR T cells [44, 45].

### Immunosuppressive tumor microenvironment

The tumor microenvironment(TME) mainly consists of numerous suppressive immune cells and molecular factors, inhibiting CAR T cells anti-tumor immune function (Figure 3B). Within the TME, T lymphocytes must overcome tremendous challenges in order to exert effective antitumor activity, including immune suppressor cells, such as Tregs, myeloid-derived suppressor cells(MDSCs), and tumor-associated macrophages (TAMs); cytokines and soluble factors associated with immunosuppression, such as TGF-β and IL-10; and checkpoint inhibitory proteins, such as PD-L1. Thus, alteration of the immunosuppressive TME may make CAR T cells restore anti-tumor effect and pave the way for improving CAR T-cell function.

Checkpoint inhibitory proteins are often upregulated on tumors, such as PD-L1 with the normal function of modulating immune response. Once PD-L1 binds to its inhibitory receptor PD-1 on CAR T cells, the function of T cells will be inhibited, or in other words, T lymphocytes will become hypofunctional. Accordingly, Suarez ER et al. made CAR T cells capable of secreting anti-PD-L1 antibodies instead of co-transfer anti-PD-L1 mAbs [46]. Besides significantly decreasing tumor growth in a humanized mouse model with renal cell carcinoma, secretion of anti-PD-L1 antibodies from CAR T cells was also able to enable human NK cells to migrate into the tumor site *in vivo*. NK cells played an anti-tumor role through ADCC as well as by providing IFNγ stimulation to CD8+ T cells. Hence, increasing the infiltration of non-T cell anti-tumor immune subsets into the TME through local antibody secretion can improve CAR T-cell therapy for solid tumors.

In contrast to PD-L1, a number of groups have demonstrated that blockade of inhibitory receptor PD-1 can augment therapy. For example, HER2 CAR T cells in combination with anti-PD-1 antibody resulted in significant tumor regression in mouse model [47]. Strikingly, we also observed that the number of MDSCs was significantly decreased in the TME of mice. Moon EK et al. studied human CAR T cells with PD-1 blockade in an immune deficient animal model, showing that PD-1 blockade using antihuman antibodies improved antitumor activity of human mesothelin-directed CARs [48]. Thus, PD-1 blockade can potently enhance CAR T-cell therapy, implying that this approach potentially make a success in patients with solid tumors.

Transforming growth factor β (TGF-β) is one of the most important inhibitory tumor cytokines. The TGF-β impairs anti-tumor responses through negative regulation of cytotoxic cell function and promotion of T-regulatory cell maturation. Thus, neutralizing TGF-β can enhance CD8+T-cell-mediated anti-tumor immune responses. In light of this effect, several approaches have been used. TGF-β receptor blockade remarkably augmented the efficacy of adoptive transfer in animal models with solid cancers [49]. To transform the tumor microenvironment, ‘armored’ CAR T cells or‘TRUCKs’ (T cells redirected for universal cytokine killing) have been investigated in pre-clinical studies. Researchers have designed these armored-CARs and TRUCKS capable of secreting pro-inflammatory cytokines in order to better function in the tumor microenvironment. Koneru M et al. have demonstrated this success in mice with human ovarian cancer xenografts [50]. CAR T cells that secrete IL-12 exhibit enhanced antitumor efficacy with increased survival, prolonged persistence of T cells, and higher systemic IFNγ.

### Specificity and safety of CAR-T cells

The vast majority of CARs recognize tumor cells in a tumor non-specific manner, in other words, CAR targets are tumor-associated antigens (TAAs) that are overexpressed on tumor cells compared to normal cells. The on-target/off-tumor effect resulting from the recognition of healthy tissues by CAR-T cells can lead to severe and even life-threatening toxicities, especially in solid tumors. Thus, strategies to improve specificity and safety of CAR T cells are urgently needed, and the implementation of effective methods to mitigate life-threatening and unexpected off-target toxicities is crucial.

Up to now, the only truly tumor-specific antigen for CAR is EGFR variant III (EGFRvIII) that is completely restricted to human cancer (most frequently observed in glioblastoma) [51, 52]. EGFRvIII CAR-T cells can precisely target tumor cells, thus both increasing the efficacy and reducing the concurrent toxicity. EGFRvIII CARs have shown promise in the treatment of glioblastoma in animal models [53, 54] and clinical trials to test the efficacy of EGFRvIII CARs in patients with glioblastoma are under way.A variety of different targeting strategies have been exploited to enhance specificity and safety of CAR T cell therapy (Figure 3C). One strategy is built on T cells modified with two different CARs, conferring CAR-T cells with the ability to differentiate tumor cells from normal cells. CAR number one contains the CD3ζ signaling domain, activating T cell function, whereas CAR number two provides the costimulation signaling function by CD28 and/or CD137 [55–57]. Full CAR T cell activation and function are only achieved in the context of the presence of both antigens.

In addition, affinity-tuned CARs offer promising opportunities with the potential to increase tumor specificity. Recently, two studies further demonstrated that CAR-T cells could distinguish tumor from normal cells expressing the same antigen at lower levels by turning the affinity of a CAR while maintaining potent antitumor efficacy *in vivo* [58, 59]. Therefore, turning sensitivity of CAR *via* scFv affinity offers a strategy to pave the way for wider use of those targets overexpressed on solid tumors for CAR-T cell therapy.

Moreover, another alternative to diminish unwanted off-target effects are inhibitory CARs (iCARs). Inhibitory CARs, specific for the antigen expressed on normal cells but not on tumor cells, will keep the normal cells from a CAR-T cell-mediated attack due to negative signaling endowed by iCARs. Fedorov et al. have demonstrated its feasibility that iCARs harnessing natural T cell inhibition exerted by PD-1 and CTLA-4 protect normal tissue from off-target effects in preclinical mouse models [60]. This success comes from checkpoint inhibition responding to an antigen found on normal tissue but not on the tumor. However, owing to iCARs failing to entirely abrogate T-cell function, further modifications such as the introduction of suicide genes [61] may contribute to removing undesirable toxicity.

### The combination of CAR-T cell therapy and other therapeutics

CAR T cells in combination with other therapies hold the potential to enhance antitumor efficacy. For instance, inhibitory receptors in the solid TME restrict the efficacy of any directly stimulatory strategy such as cytotoxic T-lymphocyte-associated antigen 4 (CTLA-4) and PD-1 [62]. Systemic administration of antagonists (blocking antibodies) to these inhibitory receptors lead to remarkable response rates in refractory solid tumors [63–65], and thus combination of blocking antibodies and CAR T cells should improve antitumor effects.

The concept of CAR T cells in combination with other drugs has opened up new therapeutic approaches. At present, most of the clinical drug treatments are prescribed without adoptive cell therapy (ACT). Thus, although there are a lot of opportunities to combine current therapies with ACT, it is important to choose a rational drug based on a deep understanding of the drug: immune system interaction. Lenalidomide, for example, has showed significant antitumor effects in patients with multiple myeloma [66]. When used with CAR T cells, it increased the infiltration of CAR T cells into the tumor site and enhanced the production and cytotoxicity of IFNγ, leading to complete cure of all treated mice [67]. All-trans retinoic acid (ATRA) can induce the differentiation of immature myeloid blasts, one of the key immunosuppressive players in the TME. Pre-clinical studies of ATRA therapy has showed that the differentiation of immunosuppressive immature myeloid cells can restore the function of anti-tumor lymphocytes [68]. when used in combination with CAR T cells targeting the GD2 antigen on osteosarcoma xenografts, both the frequency and function of tumor infiltrating MDSCs were significantly decreased, leading to an improved overall survival compared to mice treated with GD2-CAR T cells alone [69]. Therefore, this combined therapy may hold great promise in patients with solid tumors.

## FUTURE DIRECTIONS AND PERSPECTIVES

As a burgeoning therapy for neoplasm, the development of CAR T cells therapy has made nonnegligible progress, which making immune therapies for tumors enter on a new stage. The high efficiency in the treatment of hematological malignancies, especially the recurrent B lymphocyte tumor, has proved its feasibility, generating hope for patients. The success in hematological malignancies treatment encourages researchers to investigate the application of CAR T cells therapy for solid tumors. Comparing with traditional therapy for solid tumors, the CAR T cells therapy has a plenty of advantages, including superior targeting abilities, more durable curative effects and advancing at a more miraculous pace. However, there are still a number of barriers referred in this review for its application on solid tumors that we have to overcome, involving heterogenous targeting antigens expressed in solid tumors making it difficult to be targeted by specific antigens, physical immunosuppressive factors surrounding the solid tumors preventing CAR T cells from getting to solid tumors, chemokines secreted by solid tumors perturbing the direction of the correct chemotactic signals from the blood to solid tumors, tumor microenvironment destroying the efficiency of CAR T cells and the adverse effects of attacking normal tissues expressing the same antigens as solid tumors.

The final objective of the CAR T cells therapy for solid tumors is to cure solid tumors, asking for new generation cells that have the ability to survive under the tumor microenvironment and work more enduringly. For the moment, a large quantities of investigations on the CAR T cells therapy for solid tumors are underway and some clinical trials have made a little progress. In view of the current problems, varies strategies are being researched to overcome these difficulties. In the foreseeable future, it is certain that we can break down all these barriers as the improvement of experiment technologies and the appearances of more clinical data.
